# Rapamycin for longevity: the pros, the cons, and future perspectives

**DOI:** 10.3389/fragi.2025.1628187

**Published:** 2025-06-20

**Authors:** Kelley M. Roark, Philip H. Iffland

**Affiliations:** Department of Neurology, University of Maryland School of Medicine, Baltimore, MD, United States

**Keywords:** aging, sirolimus, epilepsy, everolimus, mTOR

## Abstract

Rapamycin, an antibiotic discovered in the 1970s from *Streptomyces hygroscopicus* on Easter Island (Rapanui), has become a critical tool in biomedical research. Initially recognized for its potent antifungal and immunosuppressive properties, rapamycin has recently gained significant attention for anti-aging therapy and seizure treatment via mTOR pathway inhibition. The mechanistic target of the rapamycin (mTOR) pathway is an evolutionarily conserved metabolic signaling cascade that regulates cell division, growth, and survival. There is growing evidence that mTOR pathway activity accelerates aging and the development of age-related diseases including cancer, atherosclerosis, diabetes, and declining immune function. Therefore physicians and “biohackers” are using mTOR inhibition via rapamycin (and rapamycin analogs) off-label for prevention of age-related conditions despite not being widely recognized as a treatment by the broader clinical community. Currently, rapamycin (i.e., sirolimus and everolimus) is FDA approved for the prevention of transplant organ rejection and for anti-seizure therapy in Tuberous Sclerosis Complex (TSC; caused by variants in *TSC1* or *2*). We aim to summarize the mTOR pathway, the impact rapamycin has on the mTOR pathway, and the state of rapamycin use in the field of aging and longevity. Importantly, we will discuss the gaps in knowledge, pitfalls, and potential for the use of rapamycin to prevent aging/age-related disease and discuss the lessons learned from achieving FDA approval of evirolimus for TSC-related seizures after many years of off-label use.

## Introduction

### Rapamycin in anti-aging and longevity research

Aging is defined as an intrinsic, progressive decline in physiological function that increases vulnerability to disease and death (Kirkwood, 2005; Elsevier, 2005; Vijg and Campisi, 2008; López-Otín et al., 2013). This process is characterized by cellular senescence, genomic instability, mitochondrial dysfunction, and loss of proteostasis. Researchers have long pursued interventions to delay or reverse aspects of aging and caloric restriction (CR) as a potential intervention. Initial findings demonstrated that reduced nutrient intake extended lifespan in rodents (McCay et al., 1935). Since then, CR has extended lifespans across experimental models (e.g., yeast, flies, and rodents) including with significant results in primates. However, evidence for lifespan extension by CR in humans is unclear (McCay et al., 1935; Colman et al., 2009; Mattison et al., 2012; Swindell, 2012; Longo et al., 2015; Mihaylova et al., 2023). These findings prompted the search for CR-mimetic compounds that engage similar molecular pathways without the need for chronic CR (Longo et al., 2015; Madeo et al., 2019). The effects of CR converge on nutrient-sensing pathways and therefore, the mTOR pathway and its inhibition by rapamycin has emerged as a leading CR mimetic.

mTOR complex 1 (mTORC1), the master kinase within the mTOR pathway, regulates cell growth, protein synthesis, and metabolism, and its activity increases with age, contributing to age-related pathologies (Kennedy and Lamming, 2016; Mannick and Lamming, 2023). By inhibiting mTORC1, rapamycin mimics the biochemistry of nutrient scarcity achieved by CR. Thus, suppression of mTORC1 is theorized to shift cellular activity from anabolic processes toward maintenance and repair pathways, promoting autophagy, and improved proteostasis–mechanisms associated with lifespan extension (Harrison et al., 2009; Johnson et al., 2013; Longo et al., 2015; Papadopoli et al., 2019). Translating these findings to humans remains uncertain, as the complexity of human aging and lack of validated endpoints complicate implementation (Lee et al., 2021). Some early clinical studies suggest that short-term rapamycin or analogs (rapalogs) may improve aspects of immune function in older adults (Mannick et al., 2014). However, this study relied on serologic responses to influenza vaccinations as a marker of enhanced immune function. Such markers have limited predictive value for broader immunocompetence, especially in aging populations where vaccines elicit only a weak to modest stimulus of CD8 T-cells (McElhaney et al., 2020; Riese et al., 2022). Broader measures of immunocompetence including T cell repertoire diversity, innate immune activity, and real-world infection resistance remain underexplored in human rapamycin trials (Mannick and Lamming, 2023; Lee et al., 2024). Further, the long-term effects and safety of chronic mTOR inhibition in healthy humans and whether rapamycin can truly “slow” human aging or prevent age-related diseases without unacceptable side effects is unknown.

### The mTOR pathway and the function of rapamycin

**FIGURE 1:**
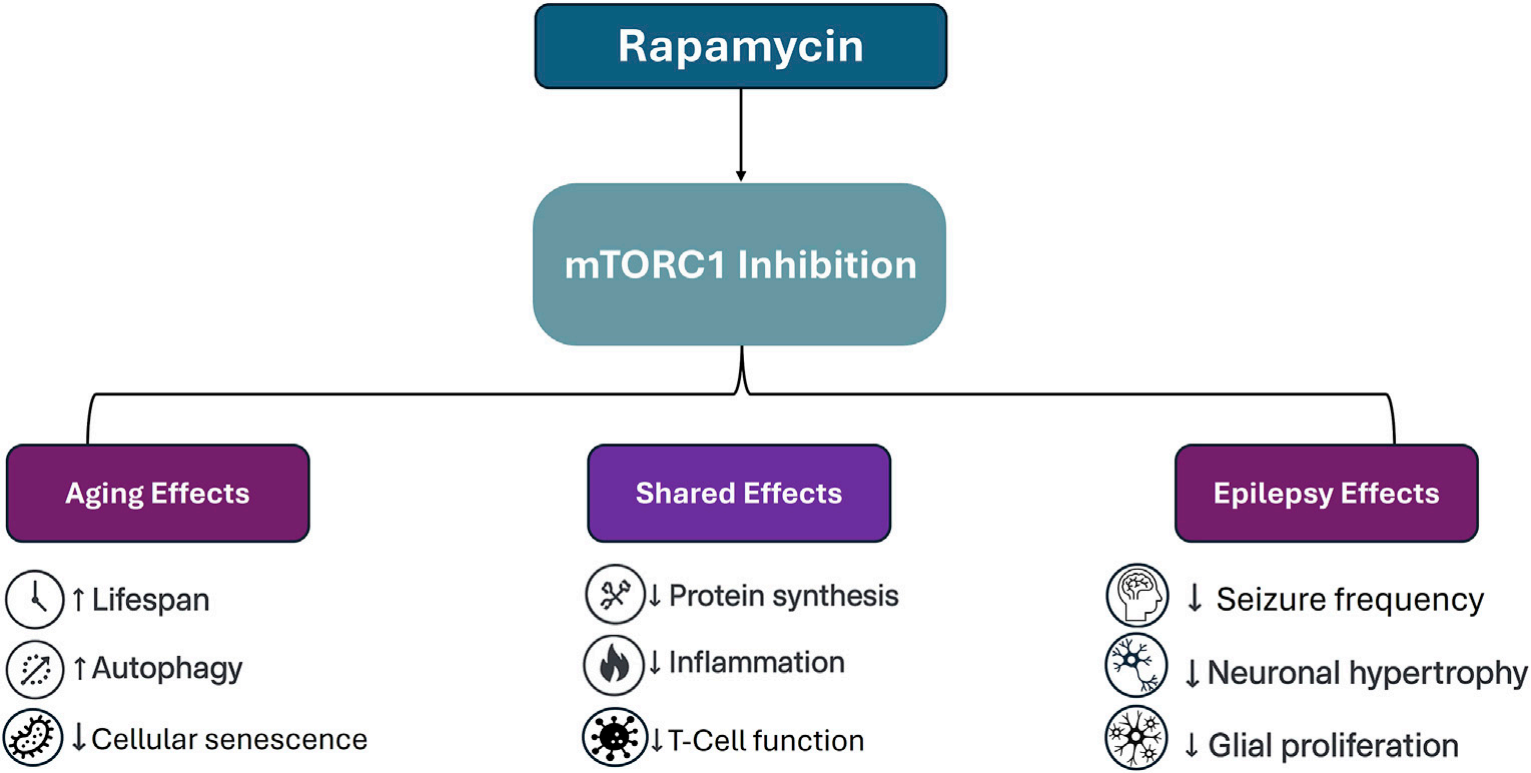
Schematic representation of rapamycin’s effects via mTORC1 inhibition across aging and epilepsy. While some outcomes such as reduced inflammation and suppressed protein synthesis are shared across both applications, others diverge significantly. In aging models, mTOR inhibition is associated with increased autophagy and delayed senescence, whereas in epilepsy, therapeutic benefits include seizure suppression and reversal of cortical hypertrophy and glial overgrowth.

The mTOR pathway was first identified through the purification of the FKBP12–rapamycin complex from mammalian cells, revealing a protein (RAFT1) homologous to yeast TOR (Target of Rapamycin) proteins (Sabatini et al., 1994). Additional discoveries in yeast identified TOR as a conserved nutrient sensing kinase, establishing the pathway’s role in regulating cell growth in response to environmental cues (Heitman et al., 1991). Together, these findings positioned the mammalian target of rapamycin (mTOR; now called “mechanistic target of rapamycin”) as a master regulator of cell growth integrating signals from growth factors, nutrients, and energy status to control protein synthesis, lipid metabolism, and autophagy (Sabatini et al., 1994; Sabatini, 2006; Saxton and Sabatini, 2017; Papadopoli et al., 2019) (Figure 1). Additional work has demonstrated mTOR’s critical role in aging and disease. Hyperactive mTOR signaling has been implicated in many age-related conditions–cancer, type 2 diabetes, neurodegeneration–and in the aging process itself (Johnson et al., 2013; López-Otín et al., 2013; Longo et al., 2015; Saxton and Sabatini, 2017). Notably, mTOR pathway activity is elevated in many tissues with age and correlates with a decline in clearance of damaged proteins and organelles (Guo et al., 2022; Dai et al., 2023; Mannick and Lamming, 2023). These observations have provided support for mTOR inhibition as a potential mechanism to slow aging. Indeed, rapamycin was the first small molecule shown to extend murine lifespan (Harrison et al., 2009).

Rapamycin’s purported geroprotective effects are often attributed to its ability to induce autophagy, a cellular recycling process responsible for degrading protein aggregates and other damage-associated molecular patterns (DAMPs) (Rubinsztein et al., 2011; Arensman and Eng, 2018; Zinecker and Simon, 2022; Szőke et al., 2023; Tabibzadeh, 2023). mTORC1 normally inhibits autophagy by phosphorylating components of the Unc-51-like autophagy-activating kinases 1 (ULK1 complex), and its inhibition by rapamycin removes this suppression and initiates autophagosome formation (Kim et al., 2011; Park et al., 2023). Online proponents of anti-aging interventions claim that rapamycin-induced autophagy promotes longevity by maintaining proteostasis and reducing “toxic burden” in post-mitotic cells which is not based in formal geroscience and lacks precise biological definition or clinical validation (Zimmermann et al., 2021; Szőke et al., 2023; Healthspan, 2025). Further, while autophagy may suppress tumor initiation by clearing damaged cellular components, it can also support the survival and growth of established tumors (Marsh et al., 2020). Thus, autophagy can suppress or enhance cancer growth depending on the cellular microenvironment and disease stage. Thus, enhancing autophagy in aging populations with elevated cancer risks and an unknown genetic background may inadvertently promote oncogenesis (Park et al., 2019; Li et al., 2020).

In addition to autophagy induction, mTOR pathway inhibition alters immune regulation through multiple mechanisms. In clinical settings, immunosuppressive mechanisms increase infection risk and impair wound healing- especially in otherwise healthy individuals without clinical manifestations that outweigh the risk of side effects (Lee et al., 2024). Indeed, both mice and humans administered rapamycin for prevention of immunoscenescense, developed glucose intolerance, hyperlipidemia, and testicular atrophy (Deutsch et al., 2007; Houde et al., 2010; Wilkinson et al., 2012). In transplant patients, long-term rapamycin caused metabolic and hematological complications (Hudson et al., 2024). These findings indicate that rapamycin may not be a universal anti-aging solution. Claims of rapamycin as a broadly applicable geroprotector should therefore be tempered by a careful evaluation of risk, mechanism, and both clinical and genetic context.

## Discussion

### Preclinical and clinical data: promises and challenges

Rapamycin administration initiated in mid-life extends lifespan by 9%–14% in mice and is associated with delayed onset of age-related pathologies (e.g., malignancies and neurodegeneration (Harrison et al., 2009; Wilkinson et al., 2012). In transgenic models predisposed to Alzheimer’s-like pathology, rapamycin prevented memory deficits and reduced cognitive decline (Spilman et al., 2010).

Rapamycin’s claimed benefits in animal models are not limited to aging but extend to models of neurological disorders. In mouse models of Tuberous Sclerosis Complex (TSC), where mTOR pathway hyperactivation is a hallmark, rapamycin prevented seizures, reduced mortality, and rescued neuropathology (i.e., glial proliferation and disrupted cortical architecture). Similar effects were observed in GATOR1-related models such as *Nprl3* knockout mice, reinforcing the potential of mTOR inhibition to alter epileptogenesis (Zeng et al., 2008; Iffland et al., 2022). Indeed, rapamycin can be effective at preventing seizures when delivered *in utero* or postnatally (Zeng et al., 2008; Parker et al., 2013; Iffland et al., 2022).

In contrast, studies on rapamycin in aging are more nuanced and context dependent. Transient, short-term rapamycin treatment in early adulthood improved late-life health outcomes in mice, extending lifespan in both sexes at low doses, but only in males at higher doses (Bitto et al., 2016). Further, late-onset rapamycin treatment in *Drosophila* did not increase lifespan, further emphasizing the temporal specificity of its effects (Schinaman et al., 2019). Interestingly, intermittent late-life administration of rapamycin has been shown to extend lifespan in both sexes, underscoring the importance of timing and dosing strategy (Arriola Apelo et al., 2016; Moel et al., 2025). The recently published PEARL trial demonstrated that low-dose intermittent rapamycin was well tolerated over 1 year and resulted in modest changes in biomarkers of biological aging, though long-term clinical benefits remain to be established. These discrepancies in lifespan extension across dosing paradigms may also depend on other factors including genetic background, dosing regimen, and timing of administration (Wilkinson et al., 2012; Bitto et al., 2016; Schinaman et al., 2019). While mTOR remains a compelling target in aging research, current animal data do not support rapamycin as a reliable intervention for extending lifespan. Thus, caution is warranted when extrapolating these findings to clinical care.

Across diverse preclinical systems, rapamycin and its analogs have some promise of delaying aging and preventing age-related diseases. While rapamycin robustly extends lifespan in nearly all murine studies (Mannick and Lamming, 2023), its translational efficacy in humans remains unclear, in part, due to the absence of standardized pharmacodynamic biomarkers. In many aging studies, surrogate biomarkers of mTORC1 inhibition, such as phosphorylated ribosomal protein S6, are either underreported or inconsistently applied, making it difficult to determine if outcomes truly reflect effective mTOR inhibition (Lee et al., 2024). Without reliable standardized biomarkers, rapamycin’s benefits remain speculative. Comparisons across studies are confounded by limited known cell-type specific differences, differences in response due to (epi)genetic background, and effects on common geriatric diseases (Mannick et al., 2014; Saxton and Sabatini, 2017; Reifsnyder et al., 2020).

A systematic review evaluated the effects of rapamycin and its derivatives on aging-related physiological changes and diseases and found improvements in the immune, cardiovascular, and integumentary systems but not in the endocrine, muscular, or neurological systems (Lee et al., 2024). Interestingly, this contrasts with robust neurological effects observed in preclinical models of epilepsy associated with mTOR pathway hyperactivating variants (“mTORopathies”) where rapamycin prevented seizures and corrected structural abnormalities (Zeng et al., 2008; Iffland et al., 2022).

The success of rapamycin in mTORopathy models stems from clearly defined molecular etiology and robust biomarkers. Specifically, highly penetrant mutations driving mTOR hyperactivation renders the pathway an actionable target. In contrast, aging is a heterogeneous and multifactorial process without a single dominant pathway, and most preclinical studies do not incorporate genetic stratification or polygenic risk scores. This may explain why mTOR inhibition yields robust disease-modifying effects in monogenic epilepsy models but produces inconsistent outcomes in aging research. Understanding rapamycin’s efficacy in epilepsy may inform how to refine translational models of aging.

However, long-term mTOR inhibition is accompanied by significant side effects. In epilepsy cohorts and transplant populations, chronic rapamycin or everolimus use is associated with mucosal ulcers, impaired wound healing, delayed tissue repair, and increased infection risk (Crino, 2016; Peterson et al., 2016; Hudson et al., 2024). Metabolic disturbances are also common, including elevated cholesterol and triglyceride levels (French et al., 2016; Lee et al., 2024). These effects are mechanistically attributed not only to mTORC1 inhibition but also the unintended suppression of mTORC2. Rapamycin induced mTORC2 inhibition has been shown to induce insulin resistance, highlighting a mechanistic trade-off between metabolic side effects and longevity benefits (Lamming et al., 2012). In female patients, hormonal side effects such as dysmenorrhea, menstrual irregularities, and ovarian dysfunction have been reported (Canpolat et al., 2018). These findings are largely derived from populations using rapamycin chronically at immunosuppressive doses. However, emerging data suggest that low-dose or intermittent rapamycin regimens may be more readily tolerated and are currently under investigation in multiple clinical trials (Kaeberlein et al., 2023; Konopka and Lamming, 2023; Mannick and Lamming, 2023; Hudson et al., 2024). Nonetheless, until long-term data are available, caution is warranted–particularly when considering the use of rapamycin in otherwise healthy individuals. The ethical implications of exposing such populations to even low levels of immunosuppression remain unresolved and merit careful deliberation and scrutiny.

Drug interactions and systemic tolerability represent key barriers to translating rapamycin’s preclinical success into routine clinical use. Rapamycin and its analogs are metabolized by cytochrome P450 enzyme CYP3A and are sensitive to pharmacokinetic interactions. Interestingly, cannabidiol (CBD) is a potent CYP3A inhibitor that increases circulating levels of mTOR inhibitors, raising the risk of toxicity (Ebrahimi-Fakhari et al., 2020; Wray et al., 2023). Given the widespread use of CBD and other cannabis products, it may be beneficial to study the interaction of these two drugs in the context of anti-aging therapy.

The clinical relevance of the above concerns was illustrated in the EXIST-3 trial, a multicenter phase III study evaluating adjunctive everolimus in patients with treatment resistant epilepsy due to TSC. Everolimus significantly reduced seizure frequency, with approximately 40% of patients in the high-dose group achieving a ≥50% reduction in seizures compared to only 15% in the placebo group (French et al., 2016). This outcome led to regulatory approval of everolimus for TSC-associated seizures. However, the EXIST-III trial also exposed the limitations of rapamycin therapy. Complete seizure freedom was rare and withdrawal of therapy frequently led to seizure recurrence indicating that mTOR inhibition is suppressive rather than curative (Sadowski et al., 2022). Importantly, adverse events including stomatitis, infections, hyperlipidemia, and cytopenias were common in the EXIST-III and other trials (Krueger et al., 2013; French et al., 2016). While these side effects were classified as “manageable” in trial settings, they may be less tolerable in individuals without overt disease who are taking rapamycin for aesthetic or anti-aging purposes. These findings offer valuable lessons for aging research: successful translation depends not only on targeting a relevant pathway but on doing so in populations where that pathway plays a central, actionable role.

### Ethical considerations regarding off-label accessibility of rapamycin

As rapamycin gains popularity for its anti-aging potential, online longevity clinics have emerged offering access to the drug with minimal medical oversight. This semi-regulated availability raises ethical concerns regarding patient safety, misinformation, and the potential for serious harm. This is best illustrated by the widely publicized case of tech entrepreneur Bryan Johnson, who undertook an elaborate self-directed anti-aging regimen involving rapamycin, metformin, and over 100 daily supplements. Despite extensive physiological tracking, Johnson ultimately discontinued rapamycin and expressed regret over its use citing side effects such as elevated blood glucose, susceptibility to infection, and impaired healing (The Economic Times, 2025). This case highlights the risks of bypassing peer-reviewed science in favor of anecdotal “biohacking” culture. Clinical literature has long documented rapamycin-associated toxicities that mirror the complaints reported by Johnson and others (Peterson et al., 2016; Hudson et al., 2024; Lee et al., 2024). The use of such a powerful immunosuppressant outside established indications, especially in otherwise healthy individuals, demands stronger ethical scrutiny and public education.

Lastly, while the FDA does not recognize aging as a disease, there is growing interest in approving therapeutics that enhance healthspan, or delay aging-related decline. However, FDA approvals are structured around specific, diagnosable indications, rather than generalized syndromes. Should rapamycin or related compounds demonstrate efficacy, they would be approved for specific indicatons (e.g., Alzhiemer’s) rather than aging *per se* under the current approval standards. Nonetheless, even within this evolving framework, it is important to note that most off-label prescribing–despite it being common clinical practice–rarely achieves FDA approval, as only about 30% of off-label prescribing is supported by adequate scientific evidence despite any clinically observed positive outcomes (Gupta and Nayak, 2014). These regulatory and evidentiary constraints must be considered when evaluating rapamycin’s future clinical and research trajectory.

### Equity, access, and ethical use in research

The rise of online rapamycin clinics has also introduced serious equity concerns. These services are often inaccessible to lower-income individuals, exacerbating existing disparities in health and longevity. While some clinics offer rapamycin for as little as $64 per month, that figure excludes substantial additional costs—membership fees typically range from $124/month to over $700 per six-month cycle, limiting access to affluent consumers *(author observations)*. As a result, the promise of rapamycin may be disproportionately realized by wealthier populations, further entrenching health inequities.

Another concern is the potential diversion of limited drug supply away from populations with approved, medically necessary, indications such as organ transplant recipients and individuals with epilepsy, TSC, and other mTORopathies. The emergence of online private pharmacies dispensing rapamycin mirrors the dynamics seen with GLP-1 agonists like semaglutide, where surging off-label use prompted both shortages and regulatory intervention. The FDA has already cracked down on unauthorized compounding and distribution of GLP-1 analogs, which may foreshadow tighter oversight of rapamycin dispensing in the future (U.S.Food and Drug Administration, 2025).

Rapamycin has been in therapeutic use for over a decade to treat seizures in individuals with variants in genes coding regulators of the mTOR pathway (Parker et al., 2013; French et al., 2016; Moloney et al., 2023). These findings underscore that rapamycin’s benefits are effective in monogenic disorders where mTOR hyperactivation is the dominant underlying pathology (Krueger et al., 2013; Iffland and Crino, 2017). These trials were predicated on pre-clinical data where phospho-S6 levels were used as a surrogate marker of mTORC1 activation to assay resected brain tissue specimens and experimental models for mTOR pathway hyperactivation (Meikle et al., 2008; Crino, 2015; Levitin et al., 2023). Unfortunately, phospho-S6 has not proven to be a consistent clinical biomarker, making it difficult to ascertain how the extent of mTOR inhibition, rapamycin blood levels, and reduction in seizures correlate. Thus, in an even more complex and dynamic biological process like aging, biomarkers will be even more challenging to deploy clinically. To begin addressing this, standard guidelines should be implemented to ensure consistency of reported data across studies. Indeed, while some studies include pharmacokinetic measurements (Mannick et al., 2014), these data are often not reported with the results, and many other studies looking at the impact of rapamycin on aging/longevity omit measurements of rapamycin levels, degree of mTOR inhibition, or other mTOR signaling-related biomarkers (Kraig et al., 2018; Mannick et al., 2018; Chung et al., 2019; Kaeberlein et al., 2023).

Another useful approach includes cross-species pharmacokinetic/pharmacodynamic (PK/PD) studies and the use of large-animal aging models to bridge the gap between murine data and human physiology. Further, trials should incorporate genetic stratification and population-specific endpoints, identifying subgroups (e.g., elderly adults with metabolic risk) most likely to benefit from intervention. Lastly, clinical trials must move beyond lifespan alone to assess validated healthspan outcomes such as immune resilience, frailty indices, and neurocognitive performance that many reveal the usefulness of rapamycin for aging-related diseases. Without these strategies, rapamycin’s promise will remain confined to experimental models, unable to meet the ethical, clinical, and scientific standards required for widespread human use.

**FIGURE 2:**
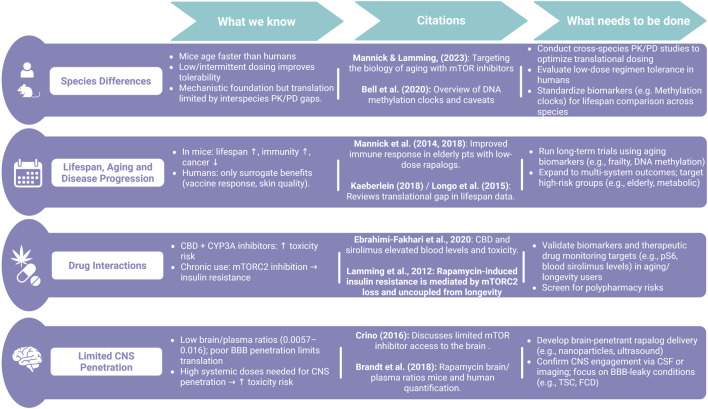
Summary of translational challenges and research gaps in applying rapamycin from animal models to humans. While preclinical studies show promising effects on lifespan and disease, translation is limited by species differences, drug interactions, poor CNS penetration, and inconsistent blood-based surrogate biomarkers that bypass established S6 phospho-targets.
